# Surgical Margins in Soft Tissue Sarcoma Management and Corresponding Local and Systemic Recurrence Rates: A Retrospective Study Covering 11 Years and 169 Patients in a Single Institution

**DOI:** 10.3390/life12111694

**Published:** 2022-10-25

**Authors:** Silvan Wittenberg, Melissa Paraskevaidis, Armin Jarosch, Anne Flörcken, Franziska Brandes, Jana Striefler, David Kaul, Siyer Roohani, Thilo Khakzad, Sven Märdian, Daniel Rau

**Affiliations:** 1 Center for Musculoskeletal Surgery, Charité–Universitätsmedizin Berlin, Corporate Member of Freie Universität Berlin and Humboldt-Universität zu Berlin, 13351 Berlin, Germany; 2 Institute of Pathology, Charité–Universitätsmedizin Berlin, Corporate Member of Freie Universität Berlin and Humboldt-Universität zu Berlin, 13351 Berlin, Germany; 3 Department of Hematology, Oncology and Cancer Immunology, Charité–Universitaätsmedizin Berlin, Corporate Member of Freie Universität Berlin and Humboldt-Universität zu Berlin, 13351 Berlin, Germany; 4 German Cancer Consortium (DKTK), Partner Site Berlin, and German Cancer Research Center (DKFZ), Charité–Universitätsmedizin Berlin, 69120 Heidelberg, Germany; 5 Center for Oncology, Department of Medicine, University Medical Center Hamburg-Eppendorf, 20251 Hamburg, Germany; 6 Department of Radiation Oncology, Charité–Universitätsmedizin Berlin, Corporate Member of Freie Universität Berlin and Humboldt-Universität zu Berlin, 13351 Berlin, Germany

**Keywords:** surgical margins, soft tissue sarcoma, local recurrence, metastases, high-grade, low-grade

## Abstract

Soft tissue sarcomas (STSs) are a diverse group of rare malignant soft tissue tumors with a high disease burden. Treatment protocols are complex and, to this day, a precise recommendation for the surgical margin width is lacking. The present study aims to analyze the width of the surgical margins in STS resection specimens and analyze them for local and systemic disease-free survival as well as for most frequent histologic STS subtypes. A total of 169 consecutive patients diagnosed and treated in curative intent in our institution following a primary and localized STS of the extremities or trunk from January 2010 to December 2020 were included in this study regardless of age. Our data reveal that low-grade STSs are best controlled locally by a surgical margin ≥2 mm and in this way also preventing distant metastases effectively. Local recurrence-free survival and metastasis-free survival in high-grade STS were improved by intact muscle fascia or periosteum at the margin when compared only to soft tissue. However, the outcome was independent of the surgical margin width, suggesting a close but negative margin may be safe in high-grade STS subtypes with less invasive growth patterns when combined with adjunct radiochemotherapy.

## 1. Introduction

Soft tissue sarcomas (STSs) is a broad term referring to a heterogenous group of rare cancers. They arise from a multitude of tissues and cells that make up the connective structure including muscle, blood vessels, nerves, and fat. Consequently, soft-tissue sarcomas can manifest anywhere in the body. Additionally, sarcomas can appear at all ages. The most recent 2020 World Health Organization (WHO) classification of soft tissue tumors identified more than 70 subtypes of STSs [1]. Nonetheless, STSs constitute less than 1% of all adult solid malignant tumors with an incidence rate of five cases or less per 100.000 adults, meeting the widely accepted criterion of a rare tumor [2,3]. Epidemiological studies identified a mean of 58 years of age at the point of diagnosis. Affected young people often exhibit an advanced stage of disease [3]. Therefore, despite its low incidence rate, STSs are accompanied by a high burden of disease and a substantial mortality and morbidity rate [4].

**Figure 1:**
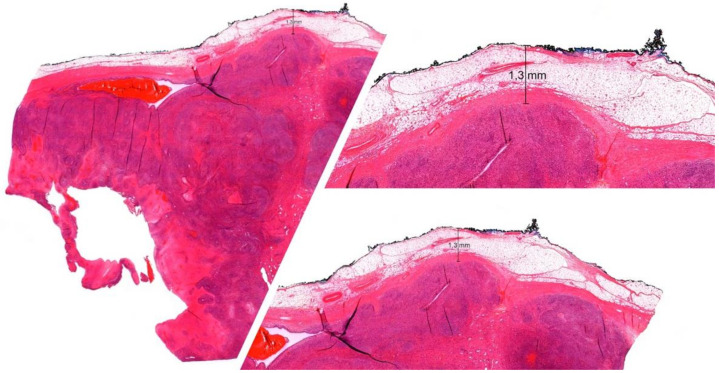
Histologic work-up of an undifferentiated pleomorphic sarcoma resection specimen in a 23-year-old female after neoadjuvant chemotherapy depicting a surgical margin width of 1.3 mm and fat tissue at the margin.

According to the current National Comprehensive Cancer Network (NCCN) and the European Society for Medical Oncology [5] guidelines, the mainstay treatment for localized high-grade STSs is surgical excision in conjunction with neoadjuvant and/or adjuvant radiotherapy and chemotherapy [5,6]. Treatment should be conducted in specialized multidisciplinary sarcoma centers on grounds of beneficial outcomes for patients and improved overall survival [7,8]. The recommendations in the literature in regard to the extent of the surgical margin of excision are vague. In low-grade STSs, some authors deem a “marginal” excision, defined as <2 cm, sufficient. On the other hand, high-grade STSs require a “wide” excision (>2 cm) to minimize the local and systemic recurrence risk [9]. However, this approximate guideline for surgical margins does not apply to all STSs. A considerable fraction of STSs (e.g., angiosarcoma, dermatofibrosarcoma, or myxofibrosarcoma) can form microscopic finger-like extensions, infiltrating the surrounding soft tissue or growing along fascial planes and altering the risk of recurrence even after surgical excision. In light of this, some authors suggest a safety margin of up to 4 cm, if feasible, in these STS subtypes [10]. Adding to the complexity of an adequate surgical margin are the distinct oncological properties of the varying tissue types (fascia, periosteum, or soft tissues) at the margin [11] (see for example Figure 1).

STSs most frequently affect the extremities, and in particular the lower extremities [12]; thus, the surgical goal should encompass complete tumor resection and consider limb preservation at the highest possible functional level. This entails preserving critical anatomic structures which in some cases might restrict a wide excision. In summary, a universally accepted definition of the width of an adequate surgical margin still does not exist. Concluding from the current literature, it might range from 10 to 40 mm in high-grade STSs [9,11,13,14,15].

This study aims to analyze the width of the closest surgical margins of STSs resection specimens performed in our institution from 2010 to 2020. We reviewed local and systemic disease-free survival in the most frequent histologic STS subtypes. The generated data may help answer the question of an adequate surgical margin in STS management.

## 2. Materials and Methods

An 11-year retrospective study was conducted including patients diagnosed with an STS that underwent curative intent treatment at our Center for Musculoskeletal Surgery from January 2010 to December 2020.

The information collected included baseline characteristics, date of diagnosis, subtype of STS, surgery performed, and adjuvant and neoadjuvant radio- and chemotherapy. A minimum of 18 months follow-up was required. All histopathological analyses were conducted by an STS pathologist. The specimens were analyzed in regard to the subtype, histologic grading, and surgical margin width.

Patients primarily treated in a different clinic, patients who first presented at a palliative (metastasized) stage, or patients with an STS recurrence needing a primary extremity ablation were excluded. Furthermore, all cases (n = 6) with insufficient documentation on surgical margin width or tissue type at the margin were also excluded.

Applying the above criteria of inclusion and exclusion, a total of 169 patients were included.

The data analyzed included age at diagnosis, gender, body mass index (BMI), American Society of Anesthesiologists (ASA) score [16], WHO STS subtype classification, tumor origin, histologic grading according to the National Federation of French Cancer Centers classification (FNCLCC) [17], tumor depth, TNM code with postoperative margin status, closest surgical margin, operating time, type of tissue at the margin, neoadjuvant and/or adjuvant therapy, regression grading according to Salzer-Kuntschik [18] after neoadjuvant chemotherapy, local or systemic recurrence, as well as time to recurrence.

The extracted data were statistically evaluated using the SPSS Statistics 28 software (International Business Machines Corporation (IBM), Armonk, New York, NY, USA).

Descriptive statistics were used to display demographic data. Nominal data were analyzed for statistical significance using the chi-squared test. Ordinal data were tested with Kruskal–Wallis test or Mann–Whitney test, depending on the number of tested groups. Ratio data were examined for normal distribution by the Shapiro–Wilk test and tested for difference using the unpaired t-test or Mann–Whitney test.

Disease-free-survival (DFS) was calculated using the univariate Kaplan–Meier method and compared with the log-rank (Mantel-Cox) technique. DFS was stratified into local recurrence-free survival (LRFS) and metastasis-free survival (MFS). LFRS and MFS were defined as time until a local or systemic recurrence was visible on CT or MRI imaging, respectively. A *p*-value < 0.05 was considered statistically significant. Regression analyses for independent risk factors were evaluated using the Cox proportional hazards model.

## 3. Results

A total of 169 patients (72 female, 97 male) with a G1-G3 STS were included in the present study. The mean age at surgery of the study population was 57.4 years (SD 18, range 2–90 years) with a mean BMI of 27.2 kg/m^2^ (SD 6.0). The mean tumor size was 107 mm (SD 79) which took 98 min (SD 83.0) on average to resect (see Table 1). The mean follow-up time was 64 months (SD 47).

**Figure 2:**
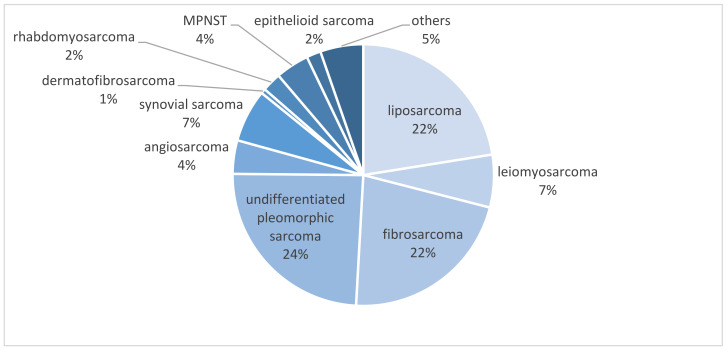
Distribution of STS subtypes in the study population.

The most frequent STS subtype was undifferentiated pleomorphic sarcoma in 24.3% of cases (41 cases), followed by liposarcoma in 22.5% (38 cases), fibrosarcoma in 21.9% (37 cases), synovial and leiomyosarcoma in 6.5% (11 cases each), angiosarcoma and MPNST in 4.1% (7 cases each), rhabdomyosarcoma in 2.4% (4 cases), epithelioid sarcoma in 1.8% (3 cases), dermatofibrosarcoma in 0.6% (1 case), and 5.3% of other not further specified soft tissue sarcomas (9 cases) (see Figure 2).

Histologic grading revealed 20.1% of low-grade (G1) sarcomas (34 cases) and 79.9% of high-grade STSs (135 cases). High-grade STSs were subclassified into G2 (52 cases, 30.8%) and G3 (83 cases, 49.1%) and treated according to current ESMO and NCCN guideline protocols with neoadjuvant and adjuvant therapy, respectively. For 16 (11.8%) patients, no adjunct therapy was received due to general patient conditions or preferences of the individual patient (see Table 2).

The neoadjuvant therapy of high-grade STSs resulted in tumor regression as was classified by Salzer-Kuntschik with a mean score of 3.53 (range 1–6, STD 1.35).

In 130 cases (76.9%), the STS localization was found on an extremity, making it the most frequent location of occurrence. Only 39 cases (23.1%) showed a manifestation on the trunk. A primary microscopically margin-negative resection (R0) was reached in 94.1% of cases (159 cases). Nine cases (5.1%) showed positive surgical margins (R1, no R2 cases). Four of these five cases were revised, and negative surgical margins could be confirmed upon revision. One resection specimen was classified as “Rx” as the surgical margin status could not be classified due to tumor fragmentation.

**Figure 3:**
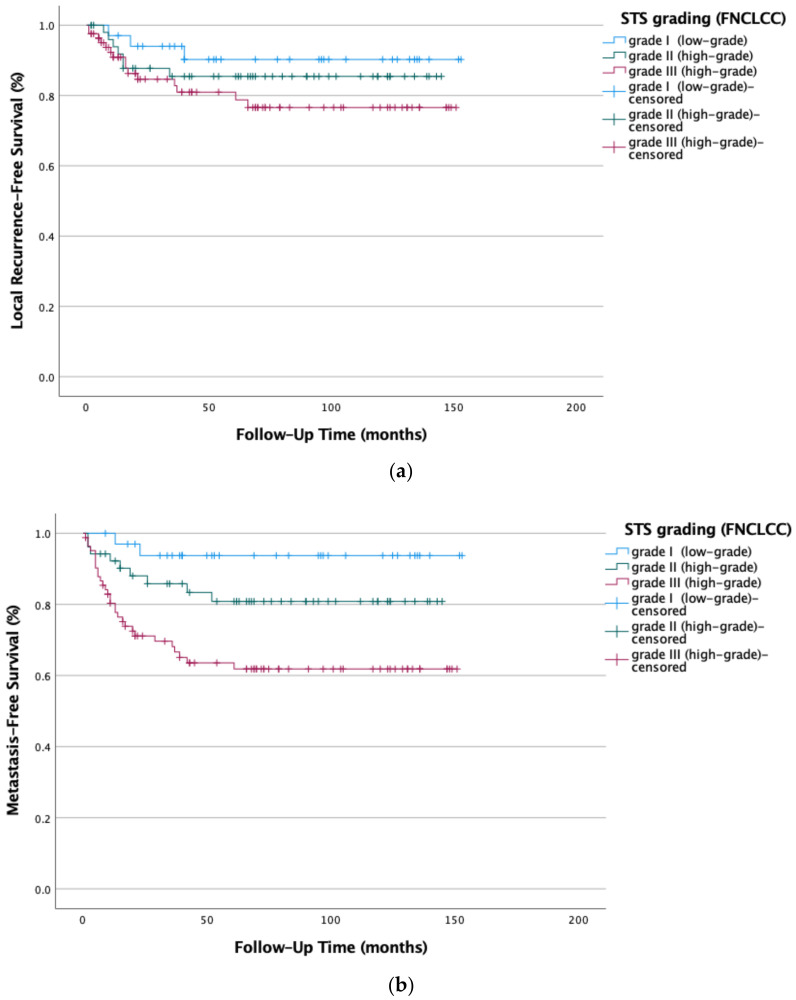
(**a**) Local recurrence-free survival (*p* = 0.096) and (**b**) metastasis-free survival (*p* = 0.01) in STSs decrease simultaneously with increased histologic grading (G1–G3); Kaplan-Meier survival functions of the study population (n = 169) stratified and plotted by their respective histologic grading (FNCLCC).

The data showed surging local recurrence rates; however, they were not statistically significant (*p* = 0.096), with higher histologic grading. The risk for distant metastases (*p* = 0.01) was significantly increased (see Figure 3a,b).

**Figure 4:**
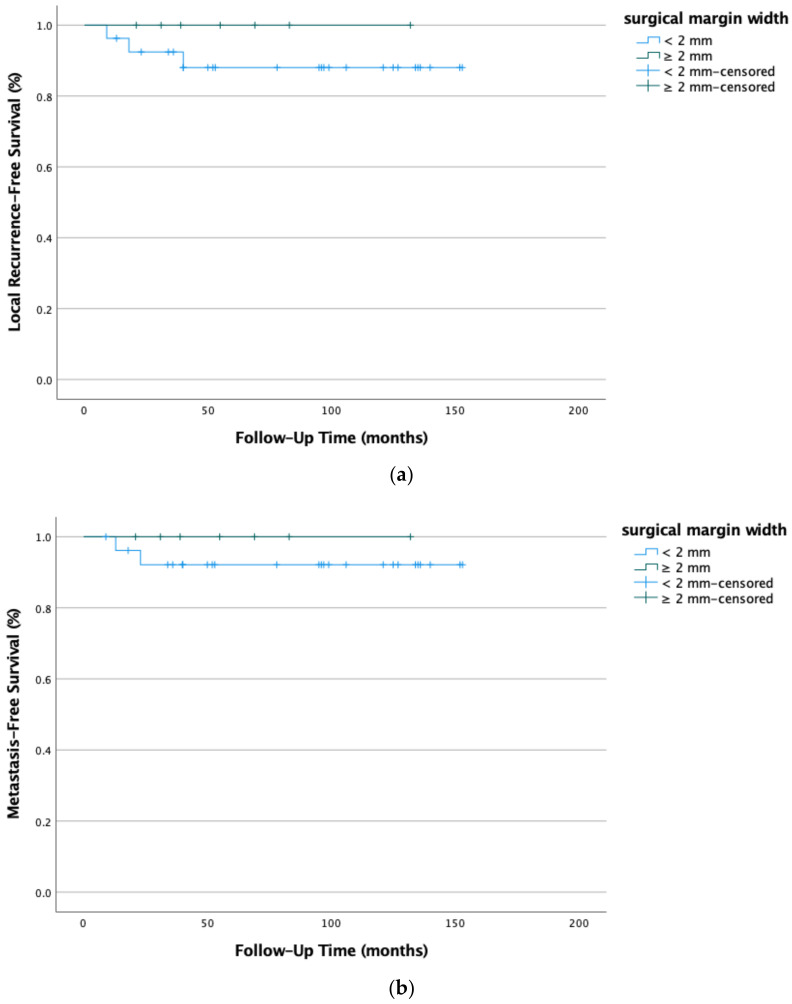
(**a**) Local recurrence-free survival (*p* = 0.393) and (**b**) metastasis-free survival (*p* = 0.471) in low-grade STSs (G1) improved if the surgical margin width was at least 2mm wide. Kaplan-Meier survival functions of low-grade STS (n = 34) stratified and plotted by surgical margin width.

Data were further tested for local LRFS and MFS in low-grade STSs in relation to surgical margin width. Low-grade STSs were usually marginally resected; the tissue type at the closest surgical margin was always soft tissue (pseudocapsule). Our data show a local recurrence rate of 8.8% and distant metastases in 5.8% of the cases with the surgical margins width ≤1 mm. Local recurrence-free survival (*p* = 0.393) and metastasis-free survival (*p* = 0.471) improved to 100% with a surgical margin width ≥2 mm. However, our findings were not statistically significant (see Figure 4a,b).

**Figure 5:**
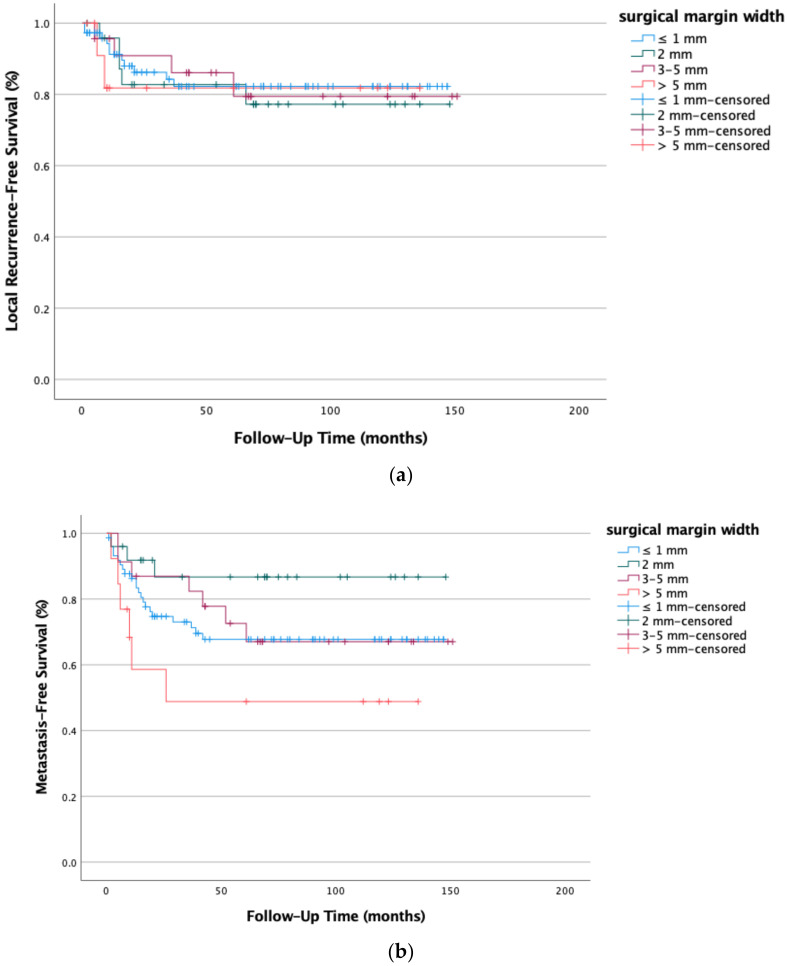
(**a**) Local recurrence-free survival (*p* = 0.727) and (**b**) metastasis-free survival (*p* = 0.471) did not improve with increased surgical margin width in high-grade STSs (G2 and G3); Kaplan-Meier survival functions of high-grade STS (n = 135) stratified and plotted by surgical margin width.

High-grade STSs were also evaluated regarding recurrence rates and surgical margin width. Local recurrence was seen in 22 cases (16.3%) and distant metastases was seen in 38 cases (28.1%)—of which eight patients had a local recurrence and distant metastases at the same time. In contrast to low-grade STSs, the surgical margin width did not play a significant role in local recurrence prevention (*p* = 0.727). On the contrary, our data even suggest increased systemic recurrence rates for cases with wide (>5 mm) surgical margins width (*p* = 0.471) (see Figure 5a,b).

**Figure 6:**
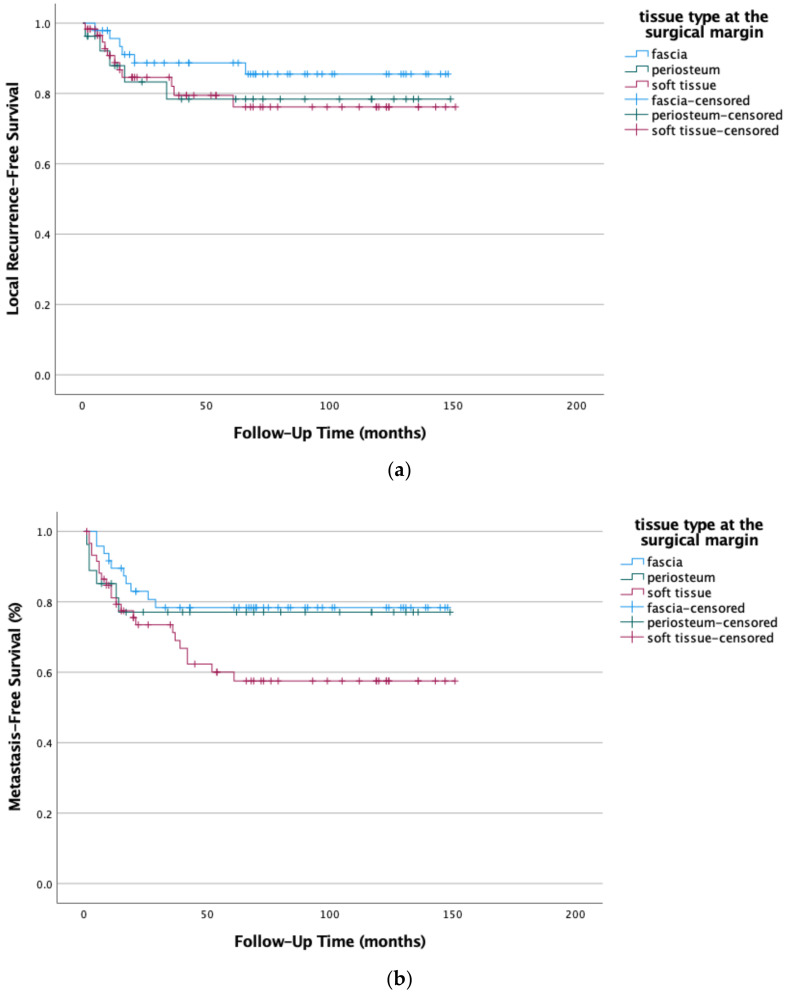
(**a**) Local recurrence-free survival (*p* = 0.273) and (**b**) metastasis-free survival (*p* = 0.046) were improved by fascia or periosteum at the surgical margin when compared to only soft tissue in high-grade STSs (G2 and G3). Kaplan-Meier survival functions of high-grade STS (n = 135) stratified and plotted by tissue type at the margin.

We examined the type of tissue bordering the slimmest surgical margin in high-grade STSs to further investigate our findings. Muscle fascia at the surgical margin showed the best local tumor control, followed by periosteum and lastly soft tissue (*p* = 0.273). In terms of distant metastases in high-grade STSs, the muscle fascia and periosteum showed almost no difference, while soft tissue was inferior to the two (*p* = 0.046) (see Figure 6a,b). Increasing the surgical margin width in cases with only soft tissue at the closest surgical margin did not improve LRFS (*p* = 0.605) or MFS (*p* = 0.952). The results indicate that the tissue type at the surgical margin is the deciding and limiting factor to prevent distant metastases rather than the margin width itself.

A multivariate Cox proportional hazards model for high-grade STSs regarding local recurrence-free and metastasis-free survival allowed us to take a closer look at other significant independent prognostic factors (see Table 3a,b). Local recurrence seems to be significantly influenced by tumor size and tumor depth. Distant metastases, in contrast, are correlated with patient age at operation and the duration of the operation, which can act as a surrogate marker for the overall invasiveness of the surgical treatment. Surprisingly, the general patient condition assessed via the ASA score as well as BMI, STS subtype, tumor location, and margin status, were not statistically relevant.

**Figure 7:**
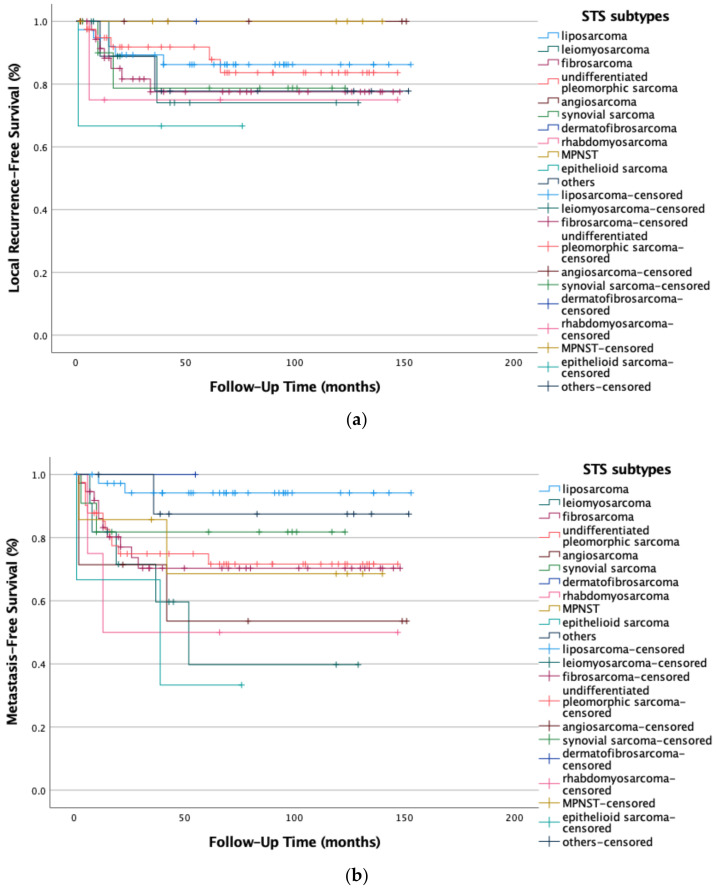
(**a**) Local recurrence-free survival (*p* = 0.861) and (**b**) metastasis-free survival (*p* = 0.052) are affected by STS (G1-G3) subtypes by a varying degree. Kaplan-Meier survival functions of all STSs (n = 169) stratified and plotted by STS subtypes.

The different STS subtypes were also tested for long-term local recurrence-free survival and metastasis-free survival regardless of histologic grading. The results show varying recurrence rates for each entity (see Figure 7a,b) which were borderline statistically significant for distant metastases (*p* = 0.052) but not for local recurrence (*p* = 0.861). Dermatofibrosarcoma protuberans, liposarcoma, and the group of not further specified STSs (others) had the best long-term disease-free systemic survival compared to the epithelioid sarcoma and leiomyosarcoma, which featured the worst outcome.

## 4. Discussion

The DFS of patients with STSs hinges on diverse factors. Among these, a microscopically negative surgical margin, the width of that margin, and the tissue type at the margin are critical and can be controlled by the surgeon to a certain extent [9,19,20].

The marginal resection of low-grade STSs along the tumor pseudocapsule, generating a surgical margin width ≥2 mm, showed an excellent local tumor control of 100% and no distant metastases. We observed a local (8.8%) and systemic (5.9%) recurrence in cases with a narrower margin width. However, it is important to note that the results did not reach statistical significance.

Equivalent studies comparing data on the safety of surgical margin width in low grade STSs are rare. The benefits of a microscopically negative surgical margin have been well studied, but only one study evaluated the local recurrence rate and safety of the surgical margin width that was sufficient in low-grade STSs. Fujiwara et al. demonstrated a 100% local tumor control in 109 low-grade STS cases with a microscopic margin ≥2 mm, and 92% for a width of 0.1–1.9 mm. Their results were highly statistically significant. Crucial to this study is that tissue specimen shrinkage between the unfixed and fixed states may translate into a 4–5 mm safety margin [21].

This study’s main takeaway is that the tissue type found at the closest surgical margin is crucial in reducing the risk of distant metastases in high-grade STSs. As outlined above, the fascia and periosteum provide the highest safety level. However, we could not show statistically improved LRFS for those tissue types when compared only to soft tissue. Furthermore, our results do not show improved outcomes with an increased margin width. This held true even if we solely considered soft tissue at the surgical margin. Wider surgical margins even suggested worse outcomes, which may be due to more aggressive surgical approaches for certain STS subtypes.

To date, literature studies on the tissue types at the surgical margins are limited. Fujiwara et al. studied 278 patients with myxofibrosarcoma and undifferentiated pleomorphic sarcoma. They concluded that a fascial or periosteal tissue margin could be translated into a margin quantity equivalent to 10 mm to prevent local recurrence [22]. Lin et al. analyzed the periosteal margin quality on bone infiltration and local recurrence rate in 50 high-grade STS cases. According to their study, periosteum is an adequate surgical margin for high-grade soft tissue sarcomas. The area of bone contact should be treated with wide excision and radiation [23].

Other authors reported similar findings on the favorable outcome of close surgical margins in high-grade STSs but did not provide further detail on the tissue type at the margin. Ahmad et al. performed a review on 382 patients with STSs on the extremities or trunk. If the patients received adjuvant radiotherapy and microscopically negative margins could be achieved, increasing the surgical margin width did not improve outcomes [24]. Harati et al. assessed 643 patients with an STS. According to their findings, microscopically negative margins but not a negative margin width were significant predictors of LFRS, MFS, and even disease-specific survival [19]. Gundle et al. examined 2217 STS cases and summarized that a negative surgical margin <1 mm may be adequate to prevent local recurrence with additional radio- and/or chemotherapy [25]. Goertz et al. examined 192 patients with undifferentiated pleomorphic sarcomas and found a negative surgical margin but not a negative margin width as a prognostic influence on LRFS and overall survival [26].

In contrast, in older case series of 111 and 279 patients, McKee et al. and Dickinson et al., respectively, showed improved local tumor control with broad negative margins of >10 mm but failed to demonstrate better overall survival rates [27,28].

Studies have been conducted to find other factors that adversely affect the possibility of living free from disease after high-grade sarcomas [19,20]. The present study found that tumor size and depth correlated significantly with LFRS. MFS significantly correlated with patient age, operating time, and histologic grading (low-grade vs. high-grade STSs). Other well-established factors such as histological subtype and margin status did not reach statistical significance. The low count of scarce STS subtypes and high percentage of primary negative margin status may contribute to this finding. We expect to detect those factors with a growing database in the future.

Some limitations in the present study need to be acknowledged. First, the study was conducted in a retrospective manner with its typical shortcomings (e.g., sampling bias). Second, a limited number of cases qualified for inclusion in the study, resulting in obvious data trends that often did not reach statistical significance if group stratification was necessary. This is especially true for rare STS subtypes, characterized by aggressive growth patterns (e.g., angiosarcoma) that require more radical surgical treatment. The findings and recommendations of this study apply only to the most common or “average” STSs undergoing further perioperative treatment in high-grade STSs. Detailed testing with higher case counts may yield different results in the future in relation to STS subtypes.

Third, it is important to note that an improvement in DFS, as in LRFS and MFS, does not automatically translate into improved disease-specific survival or overall survival. Our study was unable to obtain sufficient mortality data to test the effects of surgical margin width and tissue type on disease-specific survival and overall survival. A tumor-free status is the only benefit that may be seen on a long-term basis for the patient, so the overall long-term benefits are questionable.

## 5. Conclusions

Surgery margins ≥2 mm may be effective in controlling low-grade STSs locally and in preventing distant metastases. Further prospective and comparative trials are necessary to validate our findings.

Muscle fascia or periosteum at the margin of high-grade STSs may increase MFS and potentially LRFS. Despite this, the outcome was independent of surgical margin width, suggesting a close but negative margin may be safe in high-grade STS subtypes with less invasive growth patterns that undergo further adjunct radiochemotherapy.

## Figures and Tables

**Table 1:** Overview of the study population (number, age, ASA score), tumor size, and surgery duration.

	Total	Low-Grade STS (G1)	High-Grade STS (G2 + G3)	Sig.
Number	169	34	135	
Age	57.4 ± 18.0 years	52.8 ± 14.2 years	59.1 ± 18.6 years	**0.031**
BMI	27.2 ± 6.0 kg/m^2^	27.6 ± 8.4 kg/m^2^	27.1 ± 5.2 kg/m^2^	0.630
ASA score	2.01 ± 0.7	1.74 ± 0.7	2.1 ± 0.7	**0.009**
Tumor size	107 ± 79 mm	115 ± 74 mm	107 ± 79 mm	0.400
Operation time	98 ± 83 min	72 ± 56 min	108 ± 90 min	0.067

**Table 2:** Overview of neoadjuvant and adjuvant treatment in high-grade STSs (G2-G3).

	Applied	Not Applied
**Neoadjuvant and/or adjuvant therapy**	**119 (88.2%)**	**16 (11.8%)**
**Neoadjuvant therapy**	**82 (60.7%)**	**53 (39.3%)**
Neoadjuvant chemotherapy ± hyperthermia therapy	53 (39.3%)	82 (60.7%)
Neoadjuvant radiotherapy	6 (4.4%)	129 (95.6%)
Neoadjuvant radiochemotherapy	19 (14.1%)	116 (85.9%)
**Adjuvant therapy**	**92 (68.1%)**	**43 (31.9%)**
Adjuvant chemotherapy ± hyperthermia therapy	12 (8.9%)	123 (91.1%)
Adjuvant radiotherapy	70 (51.9%)	65 (48.1%)
Adjuvant radiochemotherapy	10 (7.4%)	125 (92.6%)

**Table 3:** (**a**) Multivariate analysis of local recurrence-free survival of independent factors in high-grade STSs (G2 and G3). (**b**) Multivariate analysis of metastasis-free survival of independent factors in high-grade STSs (G2 and G3).

(a)				
	Hazard Ratio	95.0% CI		Sig.
		Lower	Upper	
Age	1.026	0.993	1.061	0.128
ASA Score	0.642	0.293	1.405	0.267
BMI	1.051	0.949	1.165	0.338
Operating time	1.003	0.996	1.010	0.384
STS entity	1.136	0.955	1.351	0.150
STS location	1.099	0.346	3.498	0.872
STS size	1.008	1.001	1.015	** 0.035 **
STS depth	0.990	0.982	0.999	** 0.030 **
Margin status (R0)	2.464	0.565	10.740	0.230
**(b)**				
	**Hazard ratio**	**95.0% CI**		**Sig.**
		**Lower**	**Upper**	
Age	1.025	1.001	1.049	** 0.039 **
ASA Score	0.840	0.496	1.422	0.516
BMI	0.979	0.904	1.061	0.612
Operating time	1.006	1.002	1.010	** 0.002 **
STS entity	1.134	0.991	1.298	0.067
STS location	1.177	0.512	2.703	0.701
STS size	1.000	0.993	1.006	0.955
STS depth	0.998	0.991	1.005	0.612
Margin status (R0)	1.939	0.510	7.376	0.331

## Data Availability

The data presented in this study are available upon request from the corresponding author. The data are not publicly available due to strict data privacy laws in the European union.
